# Allergen-specific immunotherapy

**DOI:** 10.1186/s13223-018-0282-5

**Published:** 2018-09-12

**Authors:** William Moote, Harold Kim, Anne K. Ellis

**Affiliations:** 1 Division of Clinical Immunology & Allergy, Western University, London, ON Canada; 2 McMaster University, Hamilton, ON Canada; 3 Queen’s University, Kingston, ON Canada

## Abstract

Allergen-specific immunotherapy is a potentially disease-modifying therapy that is effective for the treatment of allergic rhinitis/conjunctivitis, allergic asthma and stinging insect hypersensitivity. However, despite its proven efficacy in these conditions, it is frequently underutilized in Canada. The decision to proceed with allergen-specific immunotherapy should be made on a case-by-case basis, taking into account individual patient factors, such as the degree to which symptoms can be reduced by avoidance measures and pharmacological therapy, the amount and type of medication required to control symptoms, the adverse effects of pharmacological treatment, and patient preferences. Since this form of therapy carries a risk of anaphylactic reactions, it should only be prescribed by physicians who are adequately trained in the treatment of allergic conditions. Furthermore, for subcutaneous therapy, injections must be given under medical supervision in clinics that are equipped to manage anaphylaxis. In this article, the authors review the indications and contraindications, patient selection criteria, and details regarding the administration, safety and efficacy of allergen-specific immunotherapy.

## Background

Allergen-specific immunotherapy is an effective treatment used by allergists and immunologists for common allergic conditions, particularly allergic rhinitis/conjunctivitis, allergic asthma and stinging insect hypersensitivity [1–7]. This form of therapy typically involves the subcutaneous administration of gradually increasing quantities of the patient’s relevant allergens until a dose is reached that is effective in inducing immunologic tolerance to the allergens. Sublingual tablet formulations are also now available in Canada for grass and ragweed allergies, as well as house dust mite-induced allergic rhinitis. These sublingual formulations involve regular self-administration of allergen extract under the tongue and do not require extensive ‘up-dosing’. The primary objectives of allergen-specific immunotherapy are to decrease the symptoms triggered by allergens and to prevent recurrence of the disease in the long-term. Currently, it is the only identified disease-modifying intervention for allergic disease [5, 6].

Despite the proven efficacy of immunotherapy for the treatment of allergic conditions, it is frequently underutilized or improperly prescribed in Canada [6, 8]. This article will review the mechanisms of immunotherapy, its indications and contraindications, patient selection criteria, and the administration, safety and efficacy of this form of therapy.

## Mechanisms of immunotherapy

Immunologic changes that occur during allergen-specific immunotherapy are complex and not completely understood. However, successful immunotherapy has been associated with a shift from T helper cell type-2 (Th2) immune responses, which are associated with the development of atopic conditions, to a better balance with more Th1 immune responses. It is also associated with the production of T regulatory cells that produce the anti-inflammatory cytokine, interleukin 10 (IL-10), amongst others such as transforming growth factor (TGF)-beta. IL-10 has been shown to reduce levels of allergen-specific immunoglobulin E (IgE) antibodies, increase levels of immunoglobulin G4 (IgG4) (“blocking”) antibodies that play a role in secondary immune responses, and reduce the release of pro-inflammatory cytokines from mast cells, eosinophils and T cells. Allergen-specific immunotherapy has also been found to decrease the recruitment of mast cells, basophils, and eosinophils to the skin, nose, eye, and bronchial mucosa after exposure to allergens, and reduce the release of mediators, such as histamine, from basophils and mast cells [5, 7]. Research surrounding the mechanisms of immunotherapy is still ongoing and will help further elucidate how this form of therapy exerts its beneficial effects in allergic diseases.

## Indications

Allergen-specific immunotherapy is indicated in patients with allergic rhinitis/conjunctivitis and/or allergic asthma who have evidence of specific IgE antibodies to clinically relevant allergens (see Table 1). It may also be effective in select patients with atopic dermatitis that is associated with aeroallergen sensitization [6, 7]. Skin prick testing (SPT) is the preferred method of testing for specific IgE antibodies. In-vitro measurement of allergen-specific IgE testing is a reasonable alternative to SPT, however, SPTs are generally considered to be more sensitive and cost effective than serum-specific IgE tests [5–7]. Patients with allergic rhinitis/conjunctivitis or allergic asthma who may be good candidates for immunotherapy include those who [7]:

**Table 1:** Allergen-specific immunotherapy: indications, contraindications and special considerations [5–7]

**Indications**	• Patients with stinging insect (venom) hypersensitivity and evidence of venom-specific IgE • Patients with allergic rhinitis/conjunctivitis and/or allergic asthma who have evidence of specific IgE antibodies to clinically relevant allergens; includes patients who: - Do not achieve control of symptoms with avoidance measures and pharmacotherapy - Do not want ongoing or long-term pharmacotherapy - Experience undesirable side effects with pharmacotherapy • Patients with atopic dermatitis associated with aeroallergen sensitization (may be considered)
**Contraindications**	• Patients with uncontrolled or severe asthma • Significant co‐morbid diseases such as cardiovascular disability • Patients on beta‐blockers (absolute contraindication with environmental allergens, relative contraindication with venoms)
**Special considerations**	• Children < 6 years of age • Pregnancy • The elderly • Patients with malignancy, immunodeficiency and autoimmune diseases

*IgE* immunoglobulin E

- have symptoms that are not well controlled by pharmacological therapy or avoidance measures;
- require high doses of medication, multiple medications, or both to maintain control of their disease;
- experience adverse effects of medications; or
- wish to avoid the long-term use of pharmacologic therapy.

Venom immunotherapy is indicated in individuals of all ages who have experienced systemic reactions to insect stings and who have specific IgE to venom allergens [9] (see Table 1). Although it is not usually recommended for patients who have had cutaneous or local reactions to insect stings, evidence suggests that venom immunotherapy significantly reduces the size and duration of large local reactions. Therefore, it may be useful in affected individuals with a history of frequent, unavoidable and/or bothersome large local reactions and detectable venom-specific IgE [9]. In addition to assessing for venom-specific IgE, consideration should also be given to measuring basal serum tryptase in patients who are candidates for venom immunotherapy since an elevated level of this serine proteinase has been shown to be an important risk factor for severe reactions before, during, and after immunotherapy [9].

Severe systemic reactions to Hymenoptera (the classification of insects that includes bees and wasps) venom are relatively uncommon, but can be fatal. The purpose of venom immunotherapy is to reduce the severity of the reactions and the risk of fatality, and to improve patient quality of life by allowing the patient to work or play outdoors without being concerned about the possibility of experiencing a serious allergic reaction [5, 9].

## Contraindications

Allergen-specific immunotherapy is contraindicated in patients with medical conditions that increase the patient’s risk of dying from treatment-related systemic reactions, such as those with severe or poorly controlled asthma or significant cardiovascular diseases (e.g., unstable angina, recent myocardial infarction, significant arrhythmia, and uncontrolled hypertension) [6, 7] (see Table 1).

Exposure to beta-blockers has been associated with more serious and treatment-resistant anaphylaxis [7, 9]. Therefore, the use of beta-blockers is an absolute contraindication to environmental allergen immunotherapy, and a relative contraindication to venom immunotherapy. In patients with life-threatening stinging insect hypersensitivity, venom immunotherapy may be considered even in those using beta-blockers because the fatal risk associated with an insect sting is far greater than the risk of an immunotherapy-related systemic reaction. Additionally, ACE inhibitors have been associated with a greater risk for more severe reactions from venom immunotherapy as well as stings [7, 9], although this finding is not consistent. Therefore, consideration should be given to discontinuing ACE inhibitors in patients undergoing venom or inhalant immunotherapy.

## Special considerations

Although there is no specific upper or lower age limit for initiating allergen-specific immunotherapy [7], special consideration should be given to its use in children under 6 years of age and the elderly. Immunotherapy is effective in children and is often well tolerated. However, children less than 6 years of age may have difficulty cooperating with the immunotherapy regimen and injections. Therefore, physicians need to weigh the risks and benefits of therapy in this patient population. The risks vs. benefits of immunotherapy also need to be considered in the elderly since these patients often have comorbid medical conditions that may increase the risk of experiencing immunotherapy-associated adverse events.

Special consideration should also be given to the use of allergen-specific immunotherapy in pregnant women, and in patients with malignancy, or immunodeficiency/autoimmune diseases (see Table 1). Immunotherapy is generally not initiated in pregnant women; however, it can be continued in women who have been on treatment prior to becoming pregnant [7, 10]. Finally, some physicians are uncomfortable manipulating the immune system in patients with autoimmune disorders, immunodeficiency syndromes, or malignant disease. However, there is no convincing evidence that allergen-specific immunotherapy is actually harmful to these patients, provided the risks and benefits of therapy have been considered [7].

## Efficacy

### Venom immunotherapy

Venom immunotherapy provides rapid protection against Hymenoptera stings, and greatly reduces the risk of systemic reactions in stinging insect-sensitive patients, with an efficacy of up to 98%. There is a residual risk of systemic reactions of approximately 5% after completion of venom immunotherapy; however, when reactions to stings do occur following completion of therapy, they are typically mild [9]. Clinical features that have been associated with a greater likelihood of relapse following the discontinuation of venom immunotherapy include: very severe reactions to a sting, systemic reactions during immunotherapy (to injections or stings), elevated basal serum tryptase levels, frequent unavoidable exposure, severe honeybee allergy, and treatment duration of less than 5 years [9].

### Allergic rhinitis

Allergen immunotherapy is an effective treatment for allergic rhinitis/conjunctivitis, particularly for patients with intermittent (seasonal) allergic rhinitis caused by pollens, including tree, grass and ragweed pollens [3, 5, 6, 11]. It has also been shown to be effective for the treatment of allergic rhinitis caused by house dust mites, Alternaria, cockroach, and cat and dog dander (although it should be noted that therapeutic doses of dog allergen are difficult to attain with the allergen extracts available in Canada). Patients’ symptoms often improve even when they were resistant to conventional drug therapy [3, 5, 12].

Evidence suggests that at least 3 years of allergen-specific immunotherapy provides beneficial effects in patients with allergic rhinitis that can persist for several years after discontinuation of therapy [13, 14]. In Canada, most allergists consider stopping immunotherapy after 5 years of adequate treatment. Recent data has made it clear that only 2 years of immunotherapy, either via the subcutaneous route or the sublingual route, is not sufficient to provide long-lasting effects [15]. Immunotherapy may also reduce the risk for the future development of asthma in children with allergic rhinitis [5].

### Asthma

Immunotherapy has been shown to be effective against allergic asthma caused by grass, ragweed, house dust mites, cat and dog dander, and Alternaria [6, 16]. A Cochrane review of 88 randomized controlled trials examining the use of allergen-specific immunotherapy in asthma management confirmed its efficacy in reducing asthma symptoms and the use of asthma medications, and improving airway hyperresponsiveness [1]. Similar benefits have been noted with sublingual immunotherapy [17], which is now available for use in Canada for grass and ragweed allergies and house dust mite-induced allergic rhinitis (see “Sublingual immunotherapy” section in this article). Evidence also suggests that allergen-specific immunotherapy may prevent the onset of asthma in atopic individuals [18, 19]. One study in children with grass and/or birch pollen allergy found that only 26% of subjects treated with immunotherapy developed asthma 3 years after completion of treatment compared to 45% who were not treated with immunotherapy [19]. Allergen-specific immunotherapy may also modify the progression of established asthma in children. A study published in the 1960s found that 70% of treated children no longer had asthma 4 years after completing immunotherapy compared to 19% of untreated control subjects, and these results were sustained up to 16 years of age [20]. However, there is no current evidence that immunotherapy influences the evolution of established asthma in adults.

### Atopic dermatitis

There is some evidence indicating that immunotherapy can be effective for atopic dermatitis when this condition is associated with aeroallergen sensitivity [6, 7]. A systematic review and meta-analysis of 8 studies involving 385 subjects found that allergen-specific immunotherapy had a significant positive effect on atopic dermatitis [21]. Therefore, immunotherapy may be considered for patients with atopic dermatitis associated with aeroallergen sensitization.

## Patient selection

The decision to proceed with allergen-specific immunotherapy should be made on a case-by-case basis, taking into account individual patient factors such as the degree to which symptoms can be reduced by avoidance measures and pharmacological therapy, the amount and type of medication required to control symptoms, and the adverse effects of pharmacological treatment [7].

Patients selected for immunotherapy should be cooperative and adherent. Those who have a history of nonadherence or who are mentally or physically unable to communicate clearly with the treating physician may be poor candidates for immunotherapy. Inability to communicate effectively with the physician will make it difficult for the patient to report signs and symptoms suggestive of systemic reactions [7].

### Venom hypersensitivity

Before deciding to proceed with venom immunotherapy, it is important to consider the natural history of venom allergy. Patients who have experienced systemic symptoms after a sting are at much greater risk of severe systemic reactions on subsequent stings compared with patients who have had only local reactions. The frequency of systemic reactions to stings ranges between 4–10% in those with a history of large local reactions compared to 25–75% in those who have had a previous systemic reaction. In general, children are at lower risk of repeated systemic reactions, as are those with a history of milder reactions [9].

It is also important to consider occupational and geographic factors that may increase the likelihood of future stings. For example, bee stings are much more common in beekeepers, their families, and their neighbours. Yellow-jacket stings are more common in certain occupations such as bakers, grocers and outdoor workers [5].

### Allergic rhinitis

**Fig. 1:**
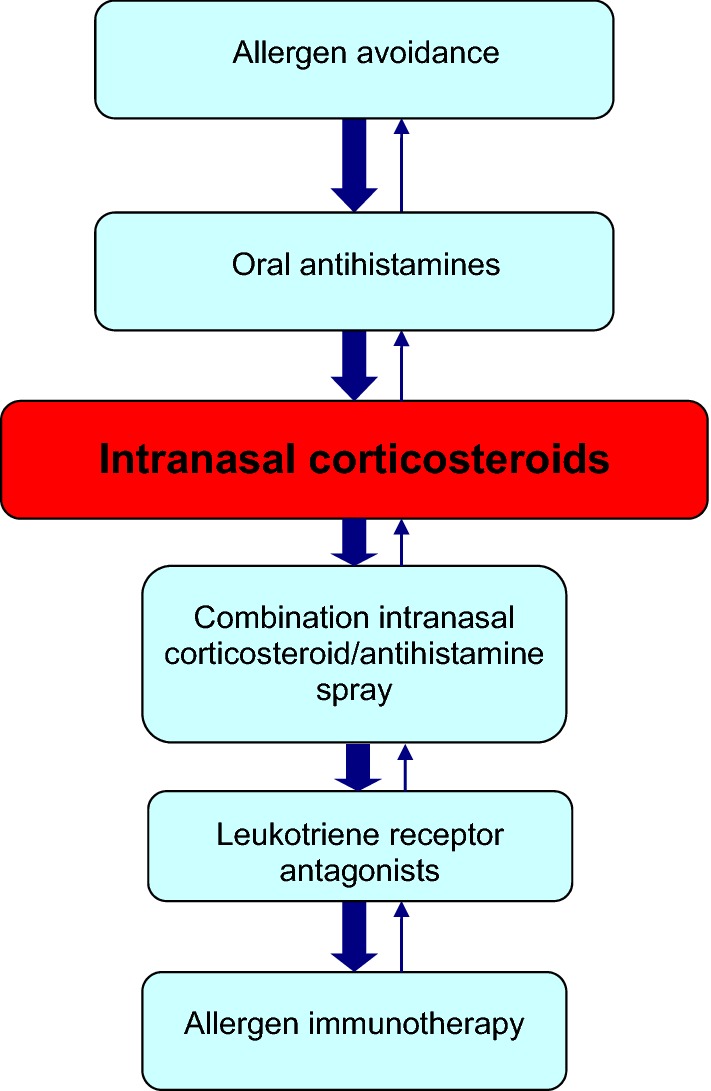
**A simplified, stepwise algorithm for the treatment of allergic rhinitis.** Note: Treatments can be used individually or in any combination

Patients with allergic rhinitis who are unable to sleep because of symptoms or whose symptoms interfere with their work or school performance despite the use of pharmacotherapy and allergen avoidance measures are particularly good candidates for immunotherapy. Those that experience adverse side effects from pharmacological therapy, such as nosebleeds from intranasal steroids or excessive drowsiness from antihistamines, and those who find pharmacotherapy inconvenient or ineffective, may also be appropriate candidates for immunotherapy [3, 5]. A flow diagram for the management of allergic rhinitis is provided in Fig. 1 (for more information on the management of allergic rhinitis, please see the *Allergic Rhinitis* article in this supplement).

### Asthma

**Fig. 2:**
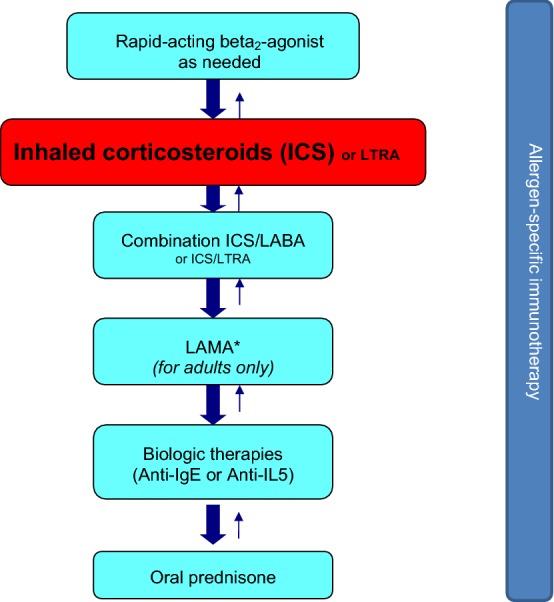
**A simplified, stepwise algorithm for the treatment of asthma.** *LAMAs are not indicated in persons < 18 years of age. *ICS* inhaled corticosteroid, *LTRA* leukotriene receptor antagonist, *LABA* long-acting beta_2_-agonist, *IgE* immunoglobulin E, *IL-5* interleukin 5, *LAMA* long-acting muscarinic receptor antagonist.** Note: Treatments can be used individually or in any combination**

As with allergic rhinitis and venom allergy, the use of allergen-specific immunotherapy in asthma should be considered on a case-by-case basis. It can be used prior to a trial of inhaled corticosteroid (ICS) therapy in patients with very mild allergic asthma and concomitant allergic rhinitis and as add-on therapy in patients using ICSs alone [16]. Allergen-specific immunotherapy may also be considered in patients using combination inhalers, ICS/leukotriene receptor antagonists (LTRAs) and/or omalizumab if asthma symptoms are controlled (see Fig. 2; for more information on the management of asthma, please see the *Asthma* article in this supplement). To reduce the risk of serious reactions, asthma symptoms must be controlled and the forced expiratory volume in 1 s (FEV_1_) should be greater than 70% predicted at the time immunotherapy is administered.

## Immunotherapy administration and schedules

Allergen-specific immunotherapy carries the risk of anaphylactic reactions (serious allergic reactions that are rapid in onset and may cause death) and, therefore, should only be prescribed by physicians who are adequately trained in the treatment of allergy and the use of immunotherapy (such as allergists and immunologists). The injections must be given where a physician is present, and in clinics that are equipped to manage possible life-threatening reactions.

Before immunotherapy is started, patients should understand its nature, benefits, risks, and costs. Counseling should also include the expected onset of efficacy and duration of treatment, as well as the risk of anaphylaxis and importance of adhering to the immunotherapy schedule. An assessment of the patient’s current health status should be made before the administration of immunotherapy injections to determine whether there have been any recent changes in the patient’s health that may require modifying or withholding treatment (e.g., uncontrolled/symptomatic asthma or exacerbation of allergy symptoms) [7].

Once it has been determined that the patient is suitable for immunotherapy, the allergist/immunologist will use extracts of clinically relevant allergens for immunotherapy treatment sets. Allergen extracts are commercially available for most of the commonly recognized allergens (e.g., grass and tree pollen, house dust mites, insect venom). When possible, standardized extracts should be utilized to prepare treatment sets since the efficacy and safety of immunotherapy are dependent on the quality of the allergen extracts used. In choosing the components for a clinically relevant allergen immunotherapy extract, the physician must also be familiar with local and regional aerobiology and indoor and outdoor allergens, paying special attention to potential allergens in the patient’s own environment. Table 2 provides the timing and concentration of common pollen and mould allergens across the various geographic regions in Canada [6].

**Table 2:** Timing and concentration of suspect pollens and mould spores in various geographic areas across Canada [6]

	Tree pollen	Grass pollen	Weed pollen	Mould spores
**British Columbia (Coastal)**	• Season: early February to mid-July • Primarily deciduous trees (alder, birch, poplar, elm, oak)	• Season: end of April/beginning of May to September • Highest grass concentrations: early June to mid-July	• Not usually a major factor; no native ragweed	• Present throughout the year except for few weeks when ground remains frozen; increase further in September and October • Most prevalent spores: Cladosporium and basidiomycetes
**British Columbia (Interior)**	• Season: late March to mid-July • Primarily deciduous trees (willow, birch, poplar)	• Season starts in early May in southern parts of the province; starts up to 1 month later in northern parts	• Ragweed is minimal	• Cladosporium can occur from April to late fall
**Prairie provinces**	• Season: first week of April until June • Primarily deciduous trees (willow, birch, poplar, alder, elm, oak, ash)	• Season: mid-May to end of September (peak is usually mid-June to early July)	• Most common: nettles and sage brush • Some ragweed, especially in Manitoba	• Can occur through spring, summer and early fall (Alternaria, Cladosporium)
**Ontario and Quebec**	• Season starts early April in southern Ontario and Quebec; may occur 4–6 weeks later in northern parts • In southern Ontario, most common are deciduous trees (birch, poplar, oak, maple, ash, elm, mulberry, willow, chestnut, hickory, Box Elder, beech, alder, walnut, sycamore, pine and juniper) • In northern Ontario, birch and poplar most common • In Quebec, ash, poplar, birch most common; maple, alder and oak are less prevalent	• Season starts mid-to-late May; a couple of weeks later in northern areas • Latter part of May and mid-June are peak seasons for grass pollination	• Ragweed season in Southern Ontario and Southwestern Quebec begins early-to-mid August • Reaches peak in late August/early September • Stops at first frost (variable) • Nettle and plantain can also contribute	• Occur during spring, summer and fall months • Concentrations may be higher late summer to fall months in Quebec • Alternaria and Cladosporium are the predominant moulds
**Maritimes & Newfoundland/Labrador**	• Season: late March to last week of June • Primarily deciduous trees (birch, poplar, alder, maple, oak, and ash)	• Season: mid-May to end of September • Peaks in early June	• Ragweed: early August to end of September	• Levels higher during the late summer and early fall months • Alternaria and Cladosporium are the predominant moulds

Typically, allergen-specific immunotherapy consists of two phases: a build-up phase (also known as up-dosing or induction) and a maintenance phase. During the build-up phase, the patient receives weekly injections, starting with a very low dose, with gradual increases in dose over the course of 3–6 months. The frequency of injections during this phase generally ranges from 1 to 3 times per week, although more rapid build-up schedules are sometimes used. After this period, the patient has usually built up sufficient tolerance to the allergen such that a maintenance (therapeutic) dose has been reached. During the maintenance phase, the patient generally receives injections of the maintenance dose every 4–6 weeks for venom and every 4 weeks for inhalant allergens, usually for a period of 3–5 years. After this period, many patients experience a prolonged, protective effect and, therefore, consideration can be given to stopping therapy, depending on risk factors for recurrence in the case of venom immunotherapy [7].

Accelerated schedules, such as rush or cluster immunotherapy, may also be used and involve the administration of several injections at increasing doses on a single visit. With cluster immunotherapy, two or three injections (at increasing doses) are given sequentially in a single day of treatment on non-consecutive days. Rush immunotherapy entails administering incremental doses of the allergen at intervals varying between 15 and 60 min over 1–3 days until the target maintenance dose is achieved. Although accelerated schedules offer the advantage of achieving the therapeutic dose much earlier than conventional immunotherapy schedules, they are also associated with an increased risk of systemic reactions [5, 7], and are not typically used in Canada for respiratory allergies. Although the safety profile of rush protocols for venom immunotherapy is good [9], these accelerated protocols may also be associated with an increased risk of systemic allergic reactions [22].

Pre-seasonal immunotherapy preparations that are administered on an annual basis are also available. They offer a much shorter build-up phase. Sublingual preparations which also require pre-seasonal treatment are also now available in Canada and are discussed in more detail below.

Patients receiving maintenance immunotherapy should be followed regularly to: assess the efficacy of treatment; monitor adverse reactions; assess patient compliance with therapy; and determine whether immunotherapy can be discontinued or if dose/schedule adjustments are required. For example, dose reductions may need to be considered during periods when the patient is exposed to increased allergen levels or when he/she is experiencing an exacerbation of symptoms.

At present, there are no specific tests or clinical markers that will distinguish between patients who will relapse and those who will remain in long-term clinical remission after discontinuing allergen immunotherapy. Therefore, the decision to continue immunotherapy beyond 3–5 years should be based on individual patient factors such as the severity of the disease, benefits sustained from treatment, reaction history, patient preference, and treatment convenience [7].

### Sublingual immunotherapy

Sublingual immunotherapy is a novel way of desensitizing patients and involves placing a tablet of allergen extract under the tongue until it is dissolved. It is currently available for the treatment of grass and ragweed allergy, as well as house dust mite-induced allergic rhinitis (with or without conjunctivitis). At present, four sublingual tablet immunotherapy products are available in Canada: Oralair^®^, Grastek^®^, Ragwitek^®^ and Acarizax™ (see Table 3) [23–26]. The sublingual route of immunotherapy offers multiple potential benefits over the subcutaneous route including the comfort of avoiding injections, the convenience of home administration, and a favourable safety profile. Like subcutaneous immunotherapy, sublingual immunotherapy is indicated for those with allergic rhinitis/conjunctivitis who have not responded to or tolerated conventional pharmacotherapy, or who are adverse to the use of these conventional treatments.

**Table 3:** Health Canada approved sublingual immunotherapy tablets [23–26]

	Extract composition	Age indication	Dose initiation	Timing of initiation before pollen season	Daily dose
Oralair^®^	Five grass pollens: • Cocksfoot • Sweet vernal grass • Rye grass • Meadow grass • Timothy	5–50 years	3 day escalation (from 100 to 300 IR)	16 weeks	300 IR
Grastek^®^	Timothy grass pollen	≥ 5 years	Full dose	At least 8 weeks	2800 BAU
Ragwitek^®^	Short ragweed pollen	18–65 years	Full dose	At least 12 weeks	12 Amb a 1-U

	Composition	Age indication	Dose initiation	Timing of initiation	Daily dose
Acarizax™	Standardized allergen extract, house dust mites (*D. farinae* and *D. pteronyssinus*)	18–65 years	Full dose	Any time during the year	1 sublingual tablet (12 SQ-HDM*) daily

*BAU* bioequivalent allergy units, *IR* index of reactivity, *U* units

*SQ-HDM is the dose unit for ACARIZAX™. SQ is a method for standardization on biological potency, major allergen content and complexity of the allergen extract. HDM is an abbreviation for house dust mite

Sublingual immunotherapy has been shown to have a sustained benefit once treatment has been discontinued, supporting its disease-modifying properties [27, 28]. While there are few studies directly comparing the efficacy of sublingual vs. subcutaneous immunotherapy, a 2013 meta-analysis that indirectly compared systematic reviews found that both forms of immunotherapy had significant benefits over placebo, however one modality could not conclusively be deemed superior to the other [29].

The most common side effects of sublingual immunotherapy are local reactions such as oral pruritus, throat irritation, and ear pruritus [6]. These symptoms typically resolve after the first week of therapy. There is a very small risk of more severe systemic allergic reactions with this type of immunotherapy and, therefore, some allergists may offer the patient an epinephrine auto-injector in case a reaction occurs at home. The risk of systemic allergic reactions is much lower with sublingual immunotherapy compared to traditional injections [6].

Similar to subcutaneous immunotherapy, sublingual immunotherapy is contraindicated in patients with severe, unstable or uncontrolled asthma and should ideally be avoided in patients on beta-blocker therapy as well as in those with active oral inflammation or sores [23–26, 30]. Sublingual immunotherapy should only be administered using the Health Canada approved products listed in Table 3.

## Safety

Subcutaneous allergen-specific immunotherapy is generally safe and well-tolerated when used in appropriately selected patients. However, local and systemic reactions may occur. Local reactions, such as redness or itching at the injection site, can generally be managed with local treatment (e.g., cool compresses or topical corticosteroids) or oral antihistamines. Systemic reactions occur in approximately 1–4% of patients on subcutaneous allergen immunotherapy [6] and can be mild to severe. The most severe reaction is anaphylaxis. Fatal anaphylactic reactions are rare, occurring in an estimated 1 in every 8 million doses of immunotherapy administered [6].

There are numerous signs and symptoms of anaphylaxis that involve the skin, gastrointestinal and respiratory tracts, and cardiovascular system (see Table 4) [31]. These symptoms typically develop within 30 min after the immunotherapy injection. In fact, most documented fatalities (73%) have occurred within 30 min of the injection [6]. It is important to note that the signs and symptoms of anaphylaxis are unpredictable and may vary from patient to patient. Therefore, the absence of one or more of the common symptoms listed in Table 4 does not rule out anaphylaxis, and should not delay immediate treatment [6]. Note that Cox et al. [32] have recently proposed a modified grading system for severe allergic reactions that may allow for more consistent reporting of these reactions and better safety comparisons across different venues and treatment protocols.

**Table 4:** Signs and symptoms of anaphylaxis [31]

Sign/symptoms	Incidence (%)
Urticaria, angioedema	87
Dyspnea	59
Dizziness, syncope	33
Diarrhea, abdominal cramps	29
Flushing	25
Upper airway edema	21
Nausea, vomiting	20
Hypotension	15
Rhinitis	8
Itch without rash	5
Seizure	1

In the event of anaphylaxis, the treatment of choice is epinephrine administered by intramuscular injection into the lateral thigh (see* Anaphylaxis* article in this supplement for more information on the diagnosis and management of anaphylaxis). Adjunctive therapies such as antihistamines, bronchodilators and systemic corticosteroids may also be used, but should never be given prior to or replace epinephrine in the treatment of anaphylaxis. In severe cases, intravenous saline or supplemental oxygen may be required [5–7].

Following a systemic reaction to immunotherapy, consideration should be given to reducing the therapeutic dose or to possibly discontinuing therapy, particularly if the patient has repeated systemic reactions following injections [5–7].

## Conclusions

Allergen-specific immunotherapy is a potentially disease-modifying therapy that is effective for the treatment of allergic rhinitis/conjunctivitis, allergic asthma and stinging insect hypersensitivity, as well as atopic dermatitis associated with aeroallergen sensitization. Although it is still unclear precisely how this form of therapy works, immunotherapy has been associated with a shift from Th2 to Th1 immune responses, and the production of T regulatory cells that dampen the immune response to relevant allergens. When used in appropriately-selected patients, allergen-specific immunotherapy is extremely safe. This form of therapy, however, does carry the risk of anaphylactic reactions and, therefore, should only be prescribed by physicians who are adequately trained in the treatment of allergy. Furthermore, immunotherapy should be administered only by physicians who are equipped to manage life-threatening anaphylaxis.

## Key take-home messages

- Allergen-specific immunotherapy is a potentially disease-modifying therapy that is effective for the treatment of allergic rhinitis/conjunctivitis, allergic asthma and stinging insect hypersensitivity, as well as atopic dermatitis associated with aeroallergen sensitivity.
- Allergen immunotherapy is contraindicated in patients with uncontrolled or severe asthma, or those with significant co-morbid cardiovascular disease.
- The use of beta-blockers is generally contraindicated for environmental allergen immunotherapy and is a relative contraindication for venom immunotherapy. Consideration of risk–benefit should be taken for concomitant use of ACE inhibitors.
- The decision to proceed with allergen immunotherapy should be made on a case-by-case basis, taking into account individual patient factors such as disease severity, efficacy of avoidance measures and pharmacological therapy, and patient preferences.
- Allergen immunotherapy carries the risk of anaphylactic reactions and, therefore, should only be prescribed by physicians who are adequately trained in the treatment of allergy.
- Injections must be given under medical supervision in clinics that are equipped to manage life-threatening anaphylaxis.
- Sublingual immunotherapy is now available in Canada for the treatment of grass and ragweed allergy, and house dust mite-induced allergic rhinitis.

## Abbreviations

Th2: T helper cell type-2
IL-10: interleukin 10
TGF: transforming growth factor
IgE: immunoglobulin E
IgG: immunoglobulin G
SPT: skin prick testing
ICS: inhaled corticosteroids
LTRAs: leukotriene receptor antagonists
FEV_1_: forced expiratory volume in 1 s
ACE: angiotensin-converting enzyme
LABA: long-acting beta_2_-agonist
IL-5: interleukin 5
LAMA: long-acting muscarinic receptor antagonist
